# *Trichoderma* controls bacterial blight caused by *Xanthomonas axonopodis* pv. *passiflorae* in *Passiflora edulis*

**DOI:** 10.3389/ffunb.2026.1920680

**Published:** 2026-09-18

**Authors:** Natália Deniz Brito, Quelmo Silva de Novaes, Rosa Hermosa, M. Belén Rubio

**Affiliations:** 1 Department of Crop Science and Animal Science, Southwest Bahia State University, Vitoria da Conquista, Bahia, Brazil; 2 Department of Microbiology and Genetics, Institute for Agribiotechnology Research (CIALE), University of Salamanca, Salamanca, Spain

**Keywords:** antibacterial activity, antioxidant enzymes, indirect biocontrol, passion fruit, reactive oxygen species, systemic defense responses

## Abstract

**Introduction:**

Several *Trichoderma* species are recognized as biocontrol agents that can enhance plant defenses against a wide range of biotic and abiotic stresses. However, few studies have investigated *Trichoderma* activity against bacterial pathogens, and the *Xanthomonas axonopodis* pv. *passiflorae (Xap)-Passiflora edulis* pathosystem remains largely unexplored.

**Methods:**

We analyzed the effects of cell-free filtrates from *Trichoderma* cultures on the growth of *Xap*. We also investigated the potential of *T. asperellum* T25 and *T. harzianum* T146, when applied to the plant growth substrate, to control bacterial blight caused by *Xap* in *P. edulis*, as well as the mechanisms underlying systemic defense responses in the host plant. Fifty-day-old plants grown in substrate treated or untreated with a *Trichoderma* strain were inoculated or not with *Xap*, and changes in the expression of marker genes representative of jasmonic acid (JA) biosynthesis (*LOX2*), salicylic acid (SA)-dependent (*PR1*), and ethylene (ET)-dependent (*PDF1.2*) defense pathways were analyzed at 48 h post-inoculation (hpi) and 14 days post-inoculation (dpi). At 14 dpi, H₂O₂ content and the activities of three antioxidant enzymes -superoxide dismutase, peroxidase, and catalase- were also evaluated.

**Results and discussion:**

*Trichoderma* filtrates showed antimicrobial activity against *Xap*. In the absence of the pathogen, strain T25 promoted the growth of *P. edulis* plants. Both *Trichoderma* strains controlled disease symptoms with similar efficacy. However, at the early stage of the *P. edulis-Xap* interaction, differential expression of SA- and JA-related genes was detected between T25-pretreated and T146-pretreated plants. Furthermore, at the later stage of the interaction (14 dpi), pretreatment with strain T146 increased ROS levels (measured as H₂O₂ content) in response to *Xap*, whereas the lowest oxidative levels were observed in plants inoculated with the pathogen alone. Our results demonstrate that *T. asperellum* and *T. harzianum* effectively control bacterial blight caused by a virulent *Xap* strain in *P. edulis*.

## Introduction

Passion fruit (*Passiflora edulis* Sims) cultivation is of high economic importance in tropical and subtropical regions, with its fruits being widely used for fresh consumption and industrial applications, including production of juices, pulps, and other processed products (Arshad et al., 2025). Beyond the food sector, bioactive compounds present in *P. edulis* have attracted interest from pharmaceutical, nutraceutical, and cosmetic industries because of their antioxidant, anti-inflammatory, and anxiolytic properties (Deng et al., 2010; Barbosa Santos et al., 2021). The global passion fruit market generated approximately US$ 3.9 billion in 2024, reflecting the growing demand for natural products and plant-based ingredients (DBMR, 2024). Brazil remains the world’s leading producer of passion fruit, accounting for nearly 70% of global production (IBGE, 2024).

Bacterial blight, caused by *Xanthomonas axonopodis* pv. *passiflorae* (*Xap*), is one of the most important diseases affecting passion fruit crops (Munhoz et al., 2015; Monzani et al., 2018). Favored by high humidity and temperature, the disease is characterized by chlorotic and necrotic lesions on leaves, branches, and fruits, frequently associated with severe defoliation, and may reduce productivity by 20-30% (Boro et al., 2011). The bacterium may also invade the vascular system and spread throughout the plant, leading to wilt and even death in susceptible cultivars (Monzani et al., 2018).

Infection by *Xanthomonas* has been shown to trigger extensive transcriptional reprogramming in rice plants, activating defense mechanisms that limit bacterial growth and disease progression (Grewal et al., 2012; Wang et al., 2019). As documented for a variety of pathogens (Torres et al., 2006), the initial plant response involves the recognition of pathogen-associated molecular patterns, which activate signaling pathways mediated by calcium fluxes, kinase cascades, and reactive oxygen species (ROS). These signals induce transcription factors that regulate defense-related genes, involving plant hormones such as jasmonic acid (JA), ethylene (ET), and salicylic acid (SA), with resistant genotypes generally displaying stronger and faster responses than susceptible plants (Grewal et al., 2012; Wang et al., 2019). A pioneering study in rice demonstrated that SA application induces systemic resistance against *Xanthomonas oryzae* pv. *oryzae* (*Xoo*) (Mohan Babu et al., 2003). Genes involved in cell wall reinforcement and antimicrobial metabolite production further contribute to resistance (Grewal et al., 2012; Wang et al., 2019). In addition, *Xanthomonas* secretes transcription activator-like effectors that modify host gene expression. Depending on the plant genotype, these effectors may either promote susceptibility or activate resistance genes, resulting in effective defense responses (Timilsina et al., 2020).

However, molecular studies on *P. edulis*-*Xap* interaction remain limited. An analysis of subtractive cDNA libraries revealed that *LOX2* is the *P. edulis* gene showing the strongest transcriptional response to *Xap* infection (Munhoz et al., 2015). Furthermore, most genes associated with canonical pathogen-recognition signaling pathways were repressed following *Xap* infection (Munhoz et al., 2015).

Management of this disease in *P. edulis* relies mainly on the use of copper-based compounds and antibiotics such as streptomycin. The effectiveness of these strategies is often temporary and limited due to the emergence of antibiotic resistance and environmental concerns (Ishida and Halfeld-Vieira, 2009; Lamichhane et al., 2018). Although breeding for host resistance remains important, pathogen variability and the instability of field resistance limit the effectiveness of this approach, highlighting the need for sustainable biocontrol alternatives (Silva et al., 2023).

Species of the genus *Trichoderma* are widely recognized as multifunctional plant-beneficial microorganisms (Woo et al., 2023). Following introduction into the soil, *Trichoderma* spp. can directly control phytopathogenic fungi through mechanisms such as mycoparasitism, competition, and antibiosis (Harman et al., 2004), as well as plant-parasitic nematodes (Martínez-Medina et al., 2017; Medeiros et al., 2017), thereby contributing to the modulation of crop-associated microbiomes and improving plant health (Illescas et al., 2020). Moreover, some *Trichoderma* strains colonize the rhizosphere (Mendoza-Mendoza et al., 2018), promoting plant growth while activating immune responses (Rivera-Méndez et al., 2020) and enhancing tolerance to abiotic stresses (Ascaso et al., 2025). Moreover, *Trichoderma* metabolites have been reported to inhibit bacterial growth (Xiao-Yan et al., 2006; Baazeem et al., 2021). Although several studies have demonstrated the biocontrol abilities of *Trichoderma* against bacterial pathogens in plants (Brotman et al., 2012; Morán-Diez et al., 2020), information regarding its biocontrol potential against *Xanthomonas* in woody plants remains scarce. Specifically, *Xap* causes severe economic losses in *P. edulis*, a high-demand crop in tropical and subtropical regions.

In the present study, we investigated the potential of two *Trichoderma* strains to control *Xap* in *P. edulis*, by analyzing the effects of fungal filtrates on bacterial growth *in vitro* and on disease severity in planta. Application of *Trichoderma* spores to the plant growth substrate enabled the assessment of systemic defense responses induced by *Trichoderma* in *P. edulis* following challenge with this bacterial pathogen. The expression of defense-related genes and the oxidative metabolism parameters were analyzed to determine the contribution of plant-mediated signals originating from *Trichoderma*-colonized roots to the priming of defense responses against *Xap*.

## Materials and methods

### Microorganisms and passion fruit seeds

Two *Trichoderma* strains, belonging to different species, were obtained from the CIALE Culture Collection (Salamanca, Spain) and used in this study: *T. asperellum* T25 (Morán-Diez et al., 2020; Pedrero-Méndez et al., 2025), and *T. harzianum* T146, isolated from the wheat rhizosphere. The strains were routinely cultured and sporulated on potato dextrose agar (PDA; Difco Laboratories, Detroit, MI, USA) at 28 °C in the dark. Conidia from 7-day-old PDA cultures were harvested, and conidial concentrations were determined as previously described by Rubio et al. (2017) for subsequent use.

The strain *Xanthomonas axonopodis* pv. *passiflorae* IBSBF2775, hereafter referred to as *Xap*, was provided by the Biological Institute of São Paulo (São Paulo, Brazil) and used as the target pathogen. *Xap* was streaked onto Luria-Bertani (LB; Invitrogen, Thermo Fisher Scientific, Waltham, MA, USA) agar plates and incubated at 30 °C for 48 h before use.

Passion fruit (*Passiflora edulis* Sims) seeds were surface-disinfected by vigorous sequential shaking in 70% ethanol and 2% sodium hypochlorite solutions for 10 min each, thoroughly washed three times with sterile distilled water, and then air-dried on a sterile gauze sheet.

### Antimicrobial *in vitro* assay

This assay was conducted to evaluate antibacterial activity of *Trichoderma* culture filtrates against *Xap*. *Trichoderma* strains were inoculated into potato dextrose broth (PDB; Difco Laboratories, Detroit, MI, USA) at a final concentration of 1 x 10^5^ spores mL^−1^ and incubated at 28 °C on a rotary shaker at 180 rpm for 72 h. The *Trichoderma* mycelium was removed by centrifugation, and the resulting supernatant was subsequently filtered through 0.22-µm pore-size membrane filters. In parallel, *Xap* was inoculated into LB medium and incubated at 30 °C and 100 rpm for 48 h. The bacterial suspension was then adjusted to a final concentration of 5 x 10^5^ cells mL^−1^.

This assay was performed in sterile 96-well microtiter plates with a final volume of 150 µL per well. *Trichoderma* cell-free filtrates were added at final concentrations of 0, 5, 10, 20 and 40% (v/v). Subsequently, 10 µL of bacterial suspension was added to each well, and the final volume was adjusted to 150 µL with LB medium. In parallel, *Xap* grown in LB medium without *Trichoderma* filtrate was included as a positive control (C+), whereas LB medium alone served as a negative control (C-).

Microtiter plates were incubated at 30 °C and 80 rpm for 24 h, and bacterial growth was determined by measuring absorbance at 620 nm (*A*_620_) using a Sunrise microtiter plate reader (Tecan Ibérica, Barcelona, Spain) after 5 s of shaking. To normalize background absorbance, the mean absorbance of the negative control (C−) was subtracted from the absorbance values of all treatments. Antibacterial activity was expressed as growth inhibition (%) relative to the positive control (C+) using the following equation: Growth inhibition (%) = [1 − (A_620_ (Treatment) − A_620_ (C−))/(A_620_ (C+) − A_620_ (C−))] × 100.

Each treatment consisted of three technical replicates using four independent fungal filtrates. Subsequently, 10 µL from each well was spread onto LB agar plates and incubated at 30 °C for 24–48 h to qualitatively confirm bacterial viability.

### Greenhouse assay

A greenhouse trial was conducted to evaluate the ability of *Trichoderma* strains T25 and T146 to protect passion fruit plants against *Xap* infection and to analyze plant defense responses.

The plant growth substrate consisted of a mixture of sandy soil, vermiculite, and peat at a 1:1:1 ratio (v/v/v), which was autoclaved twice at 121 °C and 1 atm for 60 min, with 24 h interval between the two sterilization cycles. A total of 240 pots (110 mL capacity), each containing 220 g of substrate, were prepared. *Trichoderma* conidia obtained from PDA cultures, as described above, were applied to the substrate at a final concentration of 5 x 10^6^ conidia g^−1^ substrate in a final volume of 20 mL per pot. Eighty pots were inoculated with strain T25, another 80 with strain T146, and 80 were left uninoculated as controls. Subsequently, one passion fruit seed was sown per pot, and seedlings were maintained in a growth chamber at 70% relative humidity, 28 °C, and a 12-h light/12-h dark photoperiod.

After 50 days, plant height was measured and expressed in centimeters (cm). Plants showing uniform growth (10–15 cm in height and 3–6 fully expanded leaves) were selected from each treatment. Thirty-two plants from each treatment (control, T25, and T146) were divided into two groups: 16 plants were inoculated with *Xap*, whereas the remaining 16 plants were left uninoculated. For *Xap* inoculation, the bacterium was grown in LB medium for 48 h, and the bacterial suspension was adjusted spectrophotometrically at 600 nm to an approximate concentration of 1 x 10^6^ CFU mL^−1^ using sterile 0.85% NaCl solution. Tween 20 was added at a final concentration of 0.01% (v/v) to facilitate uniform distribution on the leaf surface, and 150 mL of the resulting bacterial suspension used to spray-inoculate 48 plants.

The following treatments were established: untreated, non-inoculated control plants (C); T25-treated plants (T25); T146-treated plants (T146); plants inoculated with *Xap* alone (*Xap*); T25-treated inoculated with *Xap* (T25+*Xap*); and T146-treated inoculated with *Xap* (T146+*Xap*) plants.

At 48 h post-inoculation (hpi) with *Xap*, leaves were collected from eight plants per treatment for gene expression analysis. Four biological replicates per treatment, each consisting of pooled material from two plants, were used. Plant material was immediately frozen in liquid nitrogen and stored at -80 °C until analysis.

The remaining eight plants per treatment were maintained for disease evaluation. At 14 days post-inoculation (dpi) with *Xap*, disease incidence (DI) and disease severity (DS) were assessed using four biological replicates, each consisting of two plants. DI was calculated as the percentage of symptomatic leaves [DI = (DP × 100)/TP], where DP represents the number of leaves showing bacterial blight symptoms and TP represents the total number of evaluated leaves. DS was visually estimated according to the diagrammatic scale for bacterial leaf spot of passion fruit described by Monzani et al. (2018), based on the percentage of damaged leaf area. Following disease assessment, leaves were collected, frozen in liquid nitrogen, and stored at -80 °C for subsequent biochemical and gene expression analyses.

### Biochemical analyses of plants

H_2_O_2_ content was determined in duplicate using 50 mg of frozen plant material, following the potassium iodide method described by Velikova et al. (2000) and Illescas et al. (2021), with absorbance monitored at 390 nm. The results were expressed as µmol g^−1^ fresh weight (FW). Four biological replicates were analyzed per treatment.

Three antioxidant enzyme activities, namely superoxide dismutase (SOD), peroxidase (POD), and catalase (CAT), were determined in duplicate as previously described by Nawaz et al. (2020) and Illescas et al. (2021). Proteins were extracted by homogenizing 50 mg of frozen plant material in 1 mL of 50 mM potassium phosphate buffer (pH 7.8) followed by centrifugation at 10,000 rpm for 20 min at 4°C. The resulting supernatant was collected and protein concentration was determined according to Bradford (1976). The activity of SOD was measured according to Illescas et al. (2021) using a mixture reaction containing 2 mL of 50 mM potassium phosphate buffer (pH 7.8), 13 mM methionine, 80 μM nitro blue tetrazolium chloride (NBT), 15 μM riboflavin, and 50 μL of protein extract. One unit of SOD activity was defined as the amount of enzyme needed to inhibit the photochemical reduction of NBT by 50%. CAT and POD activities were determined spectrophotometrically as previously described by Nawaz et al. (2020), and one unit defined as the change of 0.01 absorbance unit per min. The specific activities of SOD, POD, and CAT were expressed as units min^−1^ mg^−1^ protein. Four biological replicates per treatment were analyzed.

### Quantitative real-time PCR

Total RNA was extracted from frozen plant material using TRIzol™ reagent (Invitrogen Life Technologies, Carlsbad, CA, USA) following the manufacturer’s instructions. cDNA was synthesized from 1 µg of RNA treated with DNase I (Sigma-Aldrich, St. Louis, MO, USA) and subsequently reverse-transcribed using an oligo(dT) primer and the Transcriptor First Strand cDNA Synthesis Kit (Takara, Inc., Tokyo, Japan), following the manufacturer’s protocols.

PCR reactions were performed in triplicate on a StepOnePlus thermocycler (Applied Biosystems, Foster City, CA, USA) in a total volume of 10 µL using TB Green Premix Ex Taq II (Takara) and 100 nM of each primer. The thermal cycling conditions consisted of an initial denaturation step at 95 °C for 30 s, followed by 40 cycles of denaturation at 95 °C for 5 s and annealing/extension at 60 °C for 34 s.

The expression levels of the *LOX2*, *PR1*, and *PDF1.2* genes were analyzed. Primer sequences, previously described by Munhoz et al. (2015); Yan et al. (2021), and Wang et al. (2023), are listed in **Supplementary Table 1**.

Ct values were normalized to those of the *P. edulis EF1* gene, and relative gene expression was calculated using the 2^-ΔΔCT^ method (Schmittgen and Livak, 2008). Four biological replicates were performed for each treatment.

### Statistical analysis

All statistical analyses were performed using R software version 4.4.2 (R Core Team, Vienna, Austria). Data were initially evaluated for normality of residuals and homogeneity of variances. DI, DS, and *LOX2* gene expression data were log(x + 1)-transformed prior to analysis. Experiments were conducted using a completely randomized design. Data were subjected to one-way analysis of variance (ANOVA), considering treatment as the single factor. For the *in vitro* assays with culture filtrates, each *Trichoderma* strain was analyzed separately, with filtrate concentration considered as the single factor. For the *in vivo* experiment, the six experimental treatments were considered independent levels of a single treatment factor. When significant differences were detected by ANOVA (*p* < 0.05), means were compared using Duncan’s multiple range test at the 5% significance level.

## Results

### Antimicrobial activity of *Trichoderma* against *X. axonopodis* pv. *passiflorae*

The antimicrobial activity of *Trichoderma* strains T25 and T146 against *Xap* was evaluated using an *in vitro* inhibition assay with increasing concentrations (0, 5, 10, 20, and 40%) of fungal culture filtrates (**Figure 1**). Both *Trichoderma* filtrates inhibited *Xap* growth in a concentration-dependent manner, although differences between strains were observed. Culture filtrates from strain T25 significantly reduced bacterial growth at concentrations ≥10%, whereas complete inhibition was observed at 40% (**Figure 1A**). By contrast, culture filtrates from strain T146 significantly reduced *Xap* growth at concentrations of 20% and higher, with complete inhibition also observed at 40% (**Figure 1B**). These results were further supported by qualitative growth assays on LB agar plates, in which reduced bacterial viability was observed with increasing concentrations of *Trichoderma* culture filtrates (**Figures 1C, D**). Overall, strain T25 appeared to exhibit higher direct biocontrol activity than T146.

**Figure 1:**
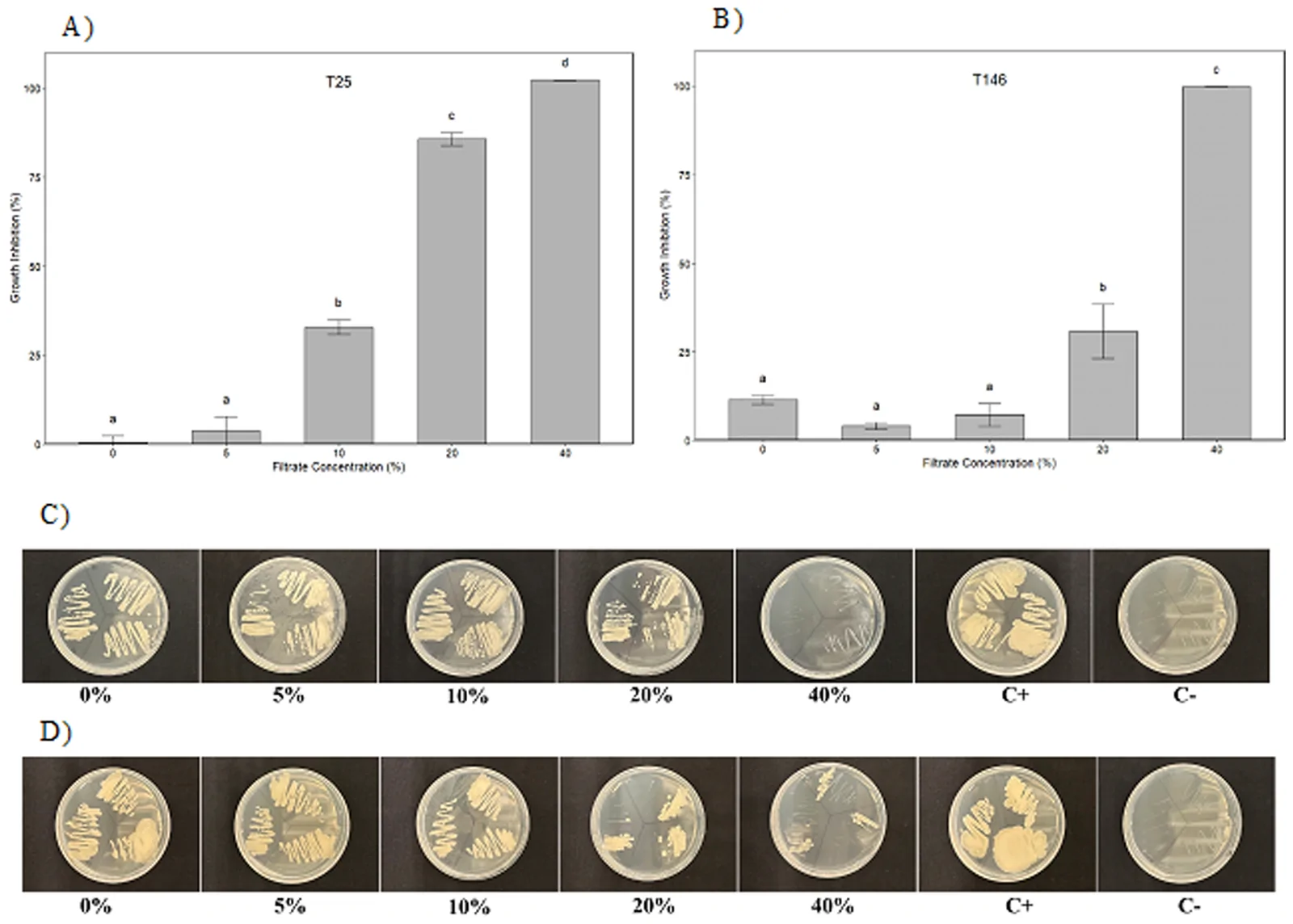
Antibacterial activity of cell-free culture filtrates from *T. asperellum* T25 **(A)** and *T. harzianum* T146 **(B)** against *X. axonopodis* pv. *passiflorae* (*Xap*), expressed as percentage of bacterial growth inhibition (%), and bacterial viability following incubation with T25 **(C)** and T146 **(D)** filtrates. Bars represent the mean ± standard deviation of four biological replicates for each treatment (n = 4). Different letters above the bars indicate significant differences according to analysis of variance (ANOVA) followed by Duncan’s test (*p* < 0.05).

### Growth-promoting effects of *T. asperellum* on *P. edulis* plants

Significant differences in plant height among T25-treated, T146-treated, and control plants were detected before inoculation with *Xap*. T25-treated plants exhibited the highest mean plant height (10.90 cm), differing significantly from both T146-treated plants and control plants (*p* < 0.05). By contrast, T146-treated and control plants showed mean heights of 8.97 and 8.43 cm, respectively, with no significant differences between them.

### Indirect biocontrol of *Trichoderma* against bacterial blight in *P. edulis* plants

A greenhouse assay was conducted to evaluate the ability of *Trichoderma* strains T25 and T146 to reduce bacterial blight caused by *Xap* in passion fruit plants. DS and DI were determined at 14 dpi with *Xap* in plants previously exposed to *Trichoderma.* T25- or T146-treated plants inoculated with *Xap* showed lower DI percentages (15.4 ± 5.0 and 18.6 ± 5.8, respectively) than plants inoculated only with the pathogen (65.7 ± 8.4), although no significant differences were observed between the two *Trichoderma* strains (**Table 1**). By contrast, plants treated with *Trichoderma* and not inoculated with *Xap* showed values comparable to those of control plants. Regarding DS, both *Trichoderma* strains reduced bacterial blight symptoms in *P. edulis* plants, resulting in DS values of 1.3 ± 0.5% and 2.0 ± 0.6% for T25+*Xap* and T146+*Xap*, respectively, compared with 11.6 ± 3.3% in plants inoculated with *Xap* alone. These severity percentages in T25+*Xap* and T146+*Xap* plants were comparable to those observed in control plants, indicating almost complete suppression of disease symptoms (**Table 1**). These results were consistent with the visual evaluation of leaves from each treatment (**Figure 2**), in which necrotic symptoms were observed only in plants inoculated with *Xap* alone.

**Table 1:** Severity and incidence of bacterial disease 14 days after *X. axonopodis* pv. *passiflorae* (*Xap*) inoculation in *P. edulis* plants pretreated with *Trichoderma* strains (*T. asperellum* T25 and *T. harzianum* T146), compared with non-pretreated, inoculated only with *Xap*, and untreated and uninoculated control plants (C).

Treatment	Incidence (%)	Severity (%)
C	0.0 ± 0.0 c	0.0 ± 0.0 b
*Xap*	65.7 ± 8.4 a	11.6 ± 3.3 a
T25	0.0 ± 0.0 c	0.0 ± 0.0 b
T146	0.0 ± 0.0 c	0.0 ± 0.0 b
T25+*Xap*	15.4 ± 5.0 b	1.3 ± 0.5 b
T146+*Xap*	18.6 ± 5.8 b	2.0 ± 0.6 b

Different letters within the same column indicate significant differences among treatments according to Duncan’s multiple range test (p < 0.05).

**Figure 2:**
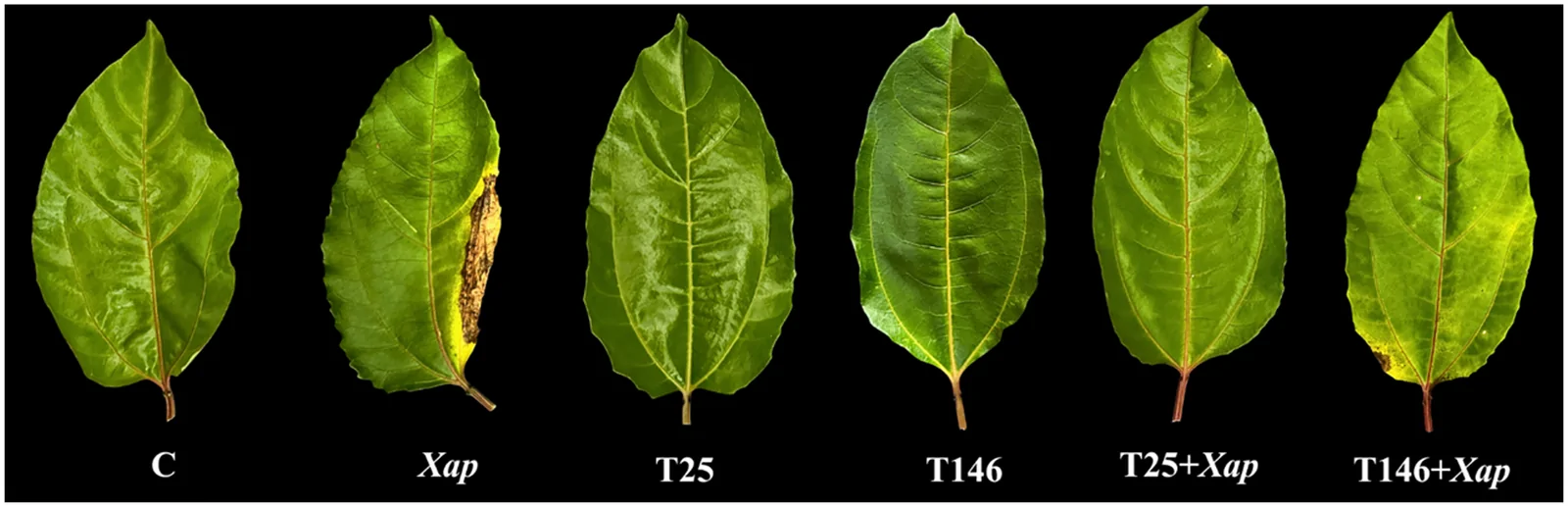
Pretreatment with *Trichoderma* strains T25 or T146 reduced bacterial blight symptoms in *P. edulis* plants. Representative leaves collected at 14 dpi with *Xap* from T25+*Xap* and T146+*Xap* plants; plants inoculated with *Xap* alone (*Xap*); plants treated with T25 or T146 without pathogen inoculation (T25 and T146); and untreated, non-inoculated control plants (C).

### Differential expression of SA- and JA pathway-related genes in *Trichoderma*-treated *P. edulis* plants in response to *X. axonopodis* pv. *passiflorae*

To evaluate the effects of *Trichoderma* strains on *P. edulis* defense responses against *Xap*, the expression of genes encoding lipoxygenase 2 (LOX2), pathogenesis-related protein 1 (PR1), and plant defensin 1.2 (PDF1.2) was analyzed at two sampling time points after pathogen inoculation in both *Trichoderma*-treated and untreated plants (**Figures 3**, **4**).

**Figure 3:**
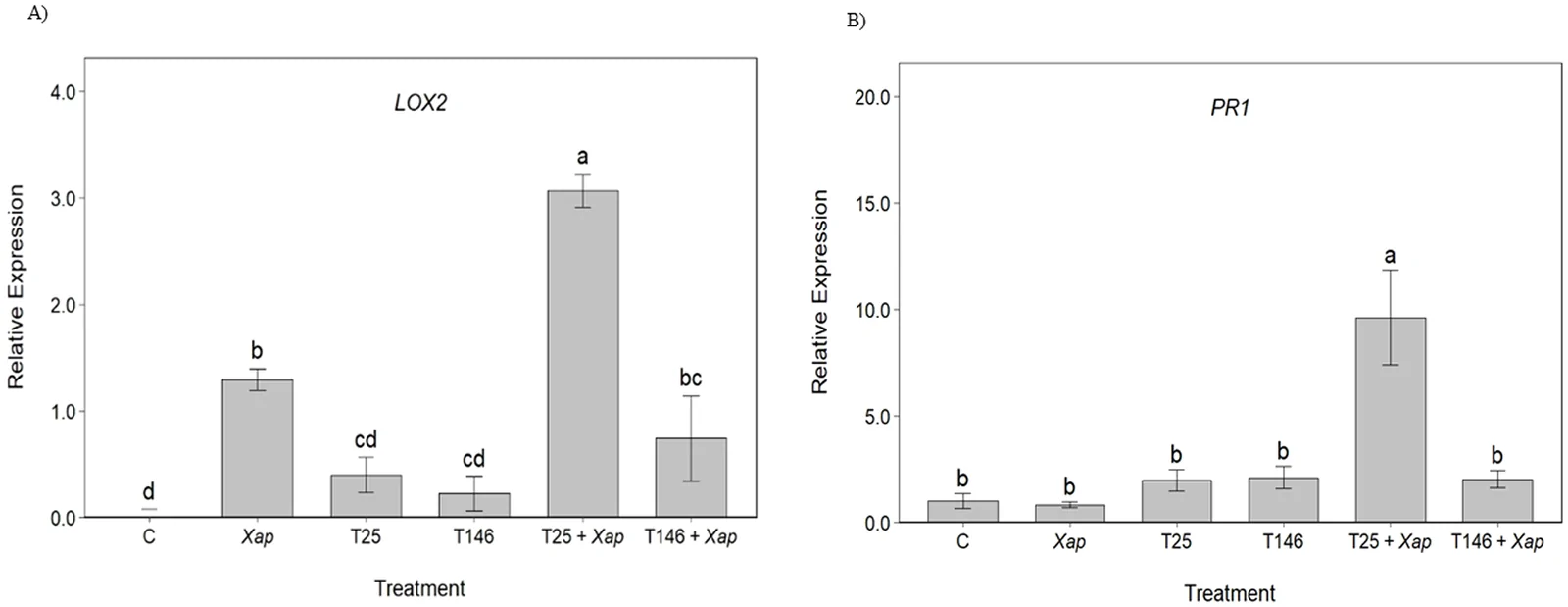
Relative expression levels of *LOX2* **(A)** and *PR1* **(B)** at 48 h post-inoculation (hpi) with *X. axonopodis* pv. *passiflorae* (*Xap*) in 60-day-old *P. edulis* plants subjected to different treatments: T25+*Xap*, T146+*Xap*, *Xap*, T25, T146, and untreated, uninoculated controls (C). Values for *PR1* represent relative expression calculated by the 2^-ΔΔCT^ method, whereas *LOX2* values are presented on a log_10_ scale. Bars represent the mean ± standard deviation of four biological replicates (n = 4). Different letters above the bars indicate significant differences according to analysis of variance (ANOVA) followed by Duncan’s test (*p* < 0.05).

**Figure 4:**
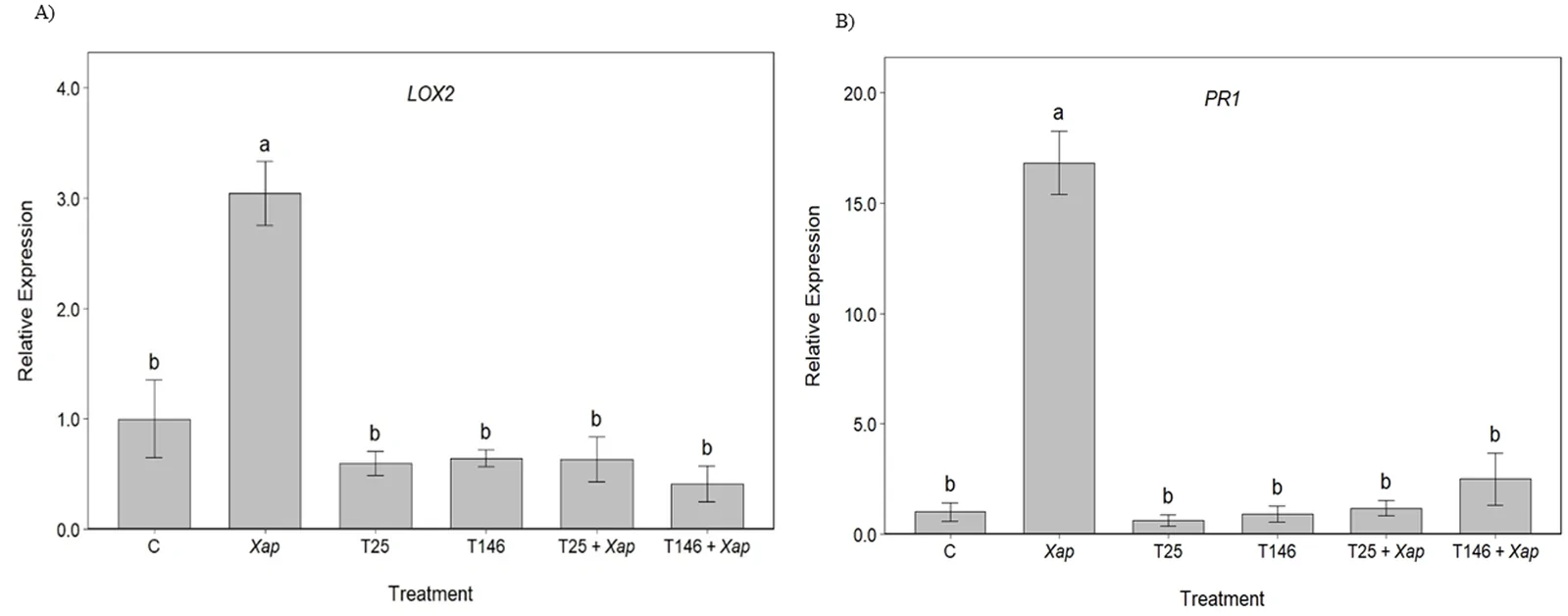
Relative expression levels of *LOX2* **(A)** and *PR1* **(B)** at 14 days post-inoculation (dpi) with *X. axonopodis* pv. *passiflorae* (*Xap*) in 60-day-old *P. edulis* plants subjected to different treatments: T25+*Xap*, T146+*Xap*, *Xap*, T25, T146, and untreated, uninoculated controls (C). Values represent relative expression calculated by the 2^-ΔΔCT^ method. Bars represent the mean ± standard deviation of four biological replicates (n = 4). Different letters above the bars indicate significant differences according to analysis of variance (ANOVA) followed by Duncan’s test (*p* < 0.05).

At 48 hpi with the pathogen, plants from the *Xap*, T25+*Xap*, and T146+*Xap* treatments showed significantly higher *LOX2* expression levels than control plants, with the highest expression observed in plants from the T25+*Xap* treatment (**Figure 3A**). Regarding *PR1* expression, upregulation gene was detected only in plants from the T25+*Xap* treatment compared with control plants (**Figure 3B**). At 14 dpi with the pathogen, plants from the *Xap* treatment exhibited upregulation of *LOX2* (**Figure 4A**) and *PR1* (**Figure 4B**), whereas no differences in gene expression were observed in plants from the remaining treatments compared with control plants. No *PDF1.2* expression was detected in plants from any of the treatments considered at either sampling time point (data not shown).

### Influence of *Trichoderma* strains on the oxidative status of *P. edulis* in response to *X. axonopodis* pv. *passiflorae*

The effect of *Trichoderma* strains on oxidative stress-related responses in *P. edulis* plants, either inoculated or uninoculated with *Xap*, was evaluated by quantifying endogenous H_2_O_2_ levels and the antioxidant enzyme activities of SOD, POD, and CAT (**Figure 5**).

**Figure 5:**
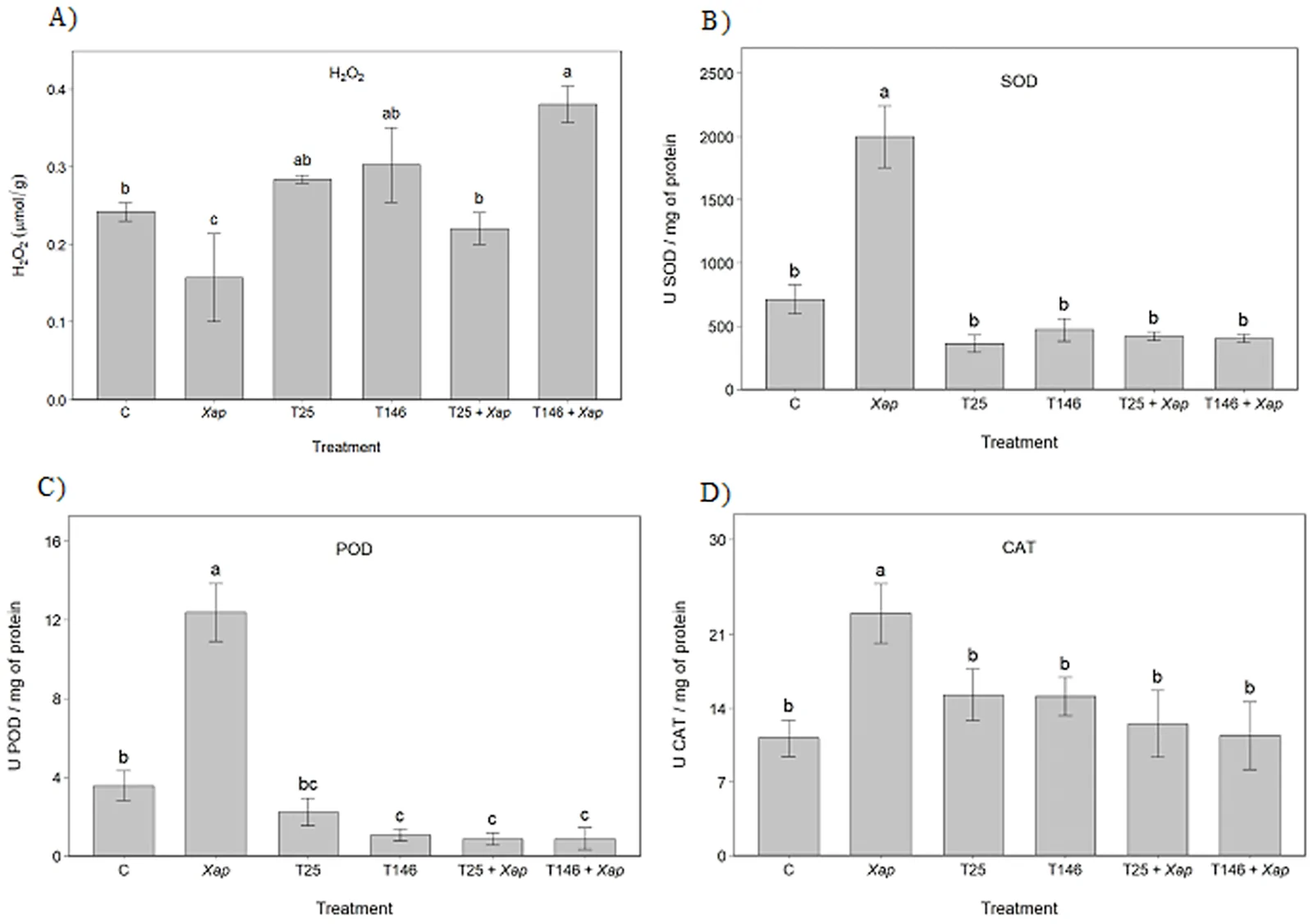
Effect of *Trichoderma* (*T. asperellum* T25 and *T. harzianum* T146) on **(A)** H_2_O_2_ content, **(B)** superoxide dismutase (SOD), **(C)** peroxidase (POD), and **(D)** catalase (CAT) activities in 64-day-old *P. edulis* plants subjected to different treatments and evaluated at 14 days of inoculation with *X. axonopodis* pv. *passiflorae* (*Xap*), when applicable. Bars represent the mean ± standard deviation of four biological replicates (n = 4). Different letters above the bars indicate significant differences according to analysis of variance (ANOVA) followed by Duncan’s test (*p* < 0.05).

Plants from the *Xap* treatment showed a significant decrease in H_2_O_2_ content (0.16 ± 0.11 μmol g^-1^ FW) compared with control plants (0.24 ± 0.02 μmol g^-1^ FW), whereas plants from the T146+*Xap* treatment exhibited the highest levels (0.38 ± 0.05 μmol g^-1^ FW) (**Figure 5A**). In parallel, the highest SOD (1,999 ± 389.88 units min^-1^ mg^-1^ protein), POD (12.40 ± 2.94 units min^-1^ mg^-1^ protein), and CAT (22.99 ± 5.63 units min^-1^ mg^-1^ protein) activity levels were detected in plants from the *Xap* treatment (**Figures 5B–D**). Moreover, only plants from the T146 (1.06 ± 0.59 units min^-1^ mg^-1^ protein), T25+*Xap* (0.86 ± 0.64 units min^-1^ mg^-1^ protein), and T146+*Xap* (0.87 ± 1.09 units min^-1^ mg^-1^ protein), treatments had significantly lower POD activity than control plants (3.57 ± 1.86 units min^-1^ mg^-1^ protein) (**Figure 5C**).

## Discussion

Managing bacterial blight in tropical and subtropical crops remains challenging under the current scenario of reducing use of fungicides and agricultural antibiotics in favor of more sustainable practices, and studies on the *P. edulis*-*Xap* pathosystem focusing on eco-friendly control alternatives are scarce. The present study demonstrated the potential of *T. asperellum* T25 and *T. harzianum* T146 as effective biocontrol agents against *Xap* in passion fruit plants, as reductions in both the incidence and severity of bacterial blight were observed in *Trichoderma*-treated plants under greenhouse conditions. *Trichoderma asperellum* and *T. harzianum* are species frequently cited in biological control studies (Woo et al., 2023), and the biocontrol potential of strain T25 against biotrophic fungi has been previously reported (Carrero-Carrón et al., 2016; Pedrero-Méndez et al., 2025). However, few studies have evaluated biocontrol activity of *Trichoderma* against bacterial pathogens, with most focusing on other plant-bacterium interactions involving phytopathogenic bacteria (Brotman et al., 2012; Morán-Diez et al., 2020; Islam et al., 2023).

Antibiosis is one of the mechanisms involved in the direct biocontrol activity of *Trichoderma* against phytopathogenic fungi (Harman et al., 2004; Woo et al., 2023). A pioneering study demonstrated that peptaibols, terpenoids, and pyrones produced by *T. koningii* exhibited antibacterial activity against a range of Gram-positive bacteria (Xiao-Yan et al., 2006). In the present study, both *Trichoderma* strains showed activity against a Gram-negative bacterium such as *Xanthomonas*, although the inhibitory effect observed with the T25 culture filtrate was markedly greater than that observed with T146. This finding is consistent with the antibacterial activity against *X. campestris* and *X. citri* reported for *T. hamatum* extracts (Baazeem et al., 2021), which was associated with the presence of 6-pentyl-α-pyrone (6-PP) as the major component. The metabolite 6-PP is a volatile organic compound frequently detected in culture extracts of *Trichoderma* spp., including *T. harzianum* (Rubio et al., 2009). Because bacterial blight caused by *Xap* is associated with severe defoliation, a reduction in pathogen inoculum in the soil resulting from the antibacterial activity of *Trichoderma* could also contribute to the effective management of this disease in passion fruit crops.

*Trichoderma* is a common soil fungus and a natural root colonizer with a recognized ability to enhance plant defenses against a wide range of pathogens (Woo et al., 2023). In the absence of the pathogen, *T. asperellum* T25 promoted the growth of *P. edulis* plants. This finding agrees with its growth-promoting effect previously reported for “Picual” olive plants (Carrero-Carrón et al., 2016). In the present study, *Trichoderma* was applied to the growth substrate of *P. edulis*, whereas *Xap* was sprayed onto the leaves. Therefore, the reduced disease incidence and severity observed in T25+*Xap* and T146+*Xap* plants appear to result from indirect biocontrol exerted by *T. asperellum* T25 and *T. harzianum* T146.

Currently, little is known about the activation defense responses by *Trichoderma* in passion fruit plants against *Xanthomonas*. At 48 hpi with the virulent bacterial strain, we observed upregulation of the *LOX2* gene in all *Xap*-inoculated plants, indicating activation of the JA-dependent pathway. Such observation is consistent with a previous report showing that the *P. edulis* gene most strongly induced in response to *Xap* attack encodes lipoxygenase 2 (Munhoz et al., 2015). Stomatal openings and wounds are important infection sites for *Xap*, and increased accumulation of transcripts encoding lipoxygenases has also been reported in passion fruit plants in response to insect herbivory (Jardim et al., 2010). In this study, T25+*Xap* plants exhibited the highest *LOX2* expression levels, accompanied by upregulation of the *PR1* gene, indicating activation of the SA-dependent pathway by *T. asperellum* T25, which has also been reported in onions challenged with a necrotrophic fungal pathogen (Rivera-Méndez et al., 2020). SA-mediated defenses play a critical role in immunity against *Xanthomonas* spp., a genus of hemibiotrophic bacterial pathogens (Durrant and Dong, 2004; Song et al., 2022).

Our findings are also consistent with a previous study demonstrating increased expression of *LOX2* and *PR1* following inoculation with *Pseudomonas syringae* pv. *tomato* (*Pst*) DC3000 in *T. asperelloides*-pretreated *Arabidopsis* plants (Brotman et al., 2012), which was associated with induction of systemic resistance against *Pst*. Similarly, *T. asperellum* has been shown to activate both SA- and JA-dependent pathways and induce resistance against *Xoo* in rice plants (Islam et al., 2023). No significant changes in *PR1* expression were detected in T146+*Xap* plants at 48 hpi with the bacterial pathogen. Nevertheless, similar reductions in disease severity were achieved in both T146+*Xap* and T25+*Xap* plants at later stages of the interaction when disease symptoms became visible. Differences among *Trichoderma* species in their capacity to confer protection against *Pst* in tomato plants have also been documented (Morán-Diez et al., 2020).

The complex role of ROS in plant defense mechanisms and their crosstalk with phytohormone signaling pathways is well established. Likewise, ROS-scavenging systems play a key role in regulating ROS generated in response to pathogen attack (Torres et al., 2006). A limitation of our study is that ROS accumulation and ROS-scavenging activities were not evaluated at early stages following pathogen challenge in passion fruit plants. To further clarify the molecular differences observed at 48 hpi in T25+*Xap* and T146+*Xap* plants compared with control plants, we analyzed expression levels of *LOX2* and *PR1*, H_2_O_2_ content, and three antioxidant enzyme activities at 14 dpi with *Xap*, when disease symptoms were already visible.

Plants inoculated only with the pathogen exhibited significantly lower H_2_O_2_ content, accompanied by significantly higher SOD, POD, and CAT activities. Interestingly, at 14 dpi, plants inoculated solely with *Xap* exhibited remarkably low H_2_O_2_ concentrations alongside the highest activities of antioxidant enzymes (SOD, CAT, and POD). This metabolic response could be explained as a survival strategy employed by the plant during advanced infection stages. Under severe and prolonged bacterial pressure, excessive ROS accumulation poses a direct threat to integrity of host cells. Thus, the host plant might shift its physiological priority toward cell survival by deploying a robust enzymatic system to actively decompose toxic H_2_O_2_, thereby preventing self-inflicted tissue necrosis at the expense of early ROS-mediated signaling. Moreover, these plants showed significantly higher expression levels of *LOX2* and *PR1*. *Xanthomonas* is known for using effector proteins to suppress basal immune responses, establish infection, and reprogram host transcription (Kay and Bonas, 2009; Timilsina et al., 2020). However, *Trichoderma* is also able to reprogram plant immunity. In this regard, *Trichoderma* has been reported to modulate JA- and SA-dependent defenses according to the nature of the pathogen infection (Martínez-Medina et al., 2017; Medeiros et al., 2017).

Enhanced transcript levels of lipoxygenase-related genes have also been reported in passion fruit plants at 5 and 9 dpi with *Xap*, together with increased SOD, CAT, and POD activities (Munhoz et al., 2015). In our study, the significantly lower H_2_O_2_ content detected in *Xap*-inoculated plants may be explained by the higher antioxidant enzyme activities observed in these plants. The discrepancy between our results and those previously reported for passion fruit plants may be attributable to differences in plant age or in the sampling time points used after *Xap* inoculation. In any case, *Xanthomonas* spp. can employ a variety of strategies to evade host defense responses (Timilsina et al., 2020).

At present, we do not have a clear explanation for the elevated expression levels of the SA-dependent defense marker gene *PR1* observed in *Xap*-inoculated plants at 14 dpi. Our results showed no significant changes in the expression of *LOX2* and *PR1* or in SOD, POD, and CAT activities in T25- and T146-treated plants compared with control plants. This may be explained by the fact that, after 2 months, *Trichoderma* colonized roots were likely already well established, and only minor systemic transcriptional changes would be expected. This interpretation is consistent with observations reported for the *T. harzianum*-tomato interaction after 3 weeks of colonization (Domínguez et al., 2016).

Likewise, neither gene expression nor antioxidant enzyme activities differed significantly in T25+*Xap* or T146+*Xap* plants relative to control plants or to plants treated only with T25 or T146. Increased phenylalanine ammonia-lyase (PAL), POD, and CAT activities have been reported following seed priming with *Trichoderma* at 72 hpi with *Xoo*, and these responses were associated with reduced disease severity in rice (Islam et al., 2023). Increased antioxidant enzyme activities and enhanced expression of *PAL* genes have also been observed in wheat plants treated with *T. asperellum* T25, which exhibited increased tolerance to drought stress (Ascaso et al., 2025).

In the absence of studies on defense regulation by *Trichoderma* in *Xap*-challenged passion fruit plants, we can only speculate that the limited defense responses observed in *Trichoderma*-treated plants are associated with indirect biocontrol. Given the low disease severity observed in *Trichoderma*-treated plants, additional defense responses may have been unnecessary. Our results demonstrate that both *Trichoderma* strains reduced bacterial disease in passion fruit plants, although differences were observed between T25+*Xap* and T146+*Xap* plants. Notably, T146+*Xap* plants differed significantly from *Xap*-inoculated plants only in H_2_O_2_ content. By contrast, activation of both SA- and JA-dependent pathways appears to contribute to defense response in *T. asperellum*-treated plants. This pattern is consistent with the undulating profile of *Trichoderma*-triggered plant defenses previously described by Rubio et al. (2014) and Woo et al. (2023). Our findings support previous suggestions that the potential of *Trichoderma* for managing bacterial diseases has been underappreciated (Islam et al., 2023; Shafi et al., 2026).

## Conclusions

Our study demonstrates that *T. asperellum* T25 and *T. harzianum* T146 exhibit direct antibacterial activity against *Xap in vitro* and effectively suppress bacterial blight development in passion fruit plants. The application of *Trichoderma* spores to the plant growth substrate significantly reduced both disease incidence and severity upon foliar challenge with *Xap*, confirming that the observed protection is mediated by the induction of systemic defense responses. At early infection stages (48 hpi), distinct expression profiles of the defense-related genes *LOX2* and *PR1* in T25-treated plants suggest that different strain-specific signaling pathways contribute to this indirect biocontrol efficacy. At later stages (14 dpi), when disease symptoms were fully developed, plants inoculated with *Xap* alone exhibited the lowest H_2_O_2_ levels alongside the highest antioxidant enzymatic activities (SOD, POD, CAT) and the strongest upregulation of defense genes (*LOX2*, *PR1*). This indicates a higher physiological demand for late-stage defense activation in plants lacking *Trichoderma* priming. Overall, our findings highlight the strong potential of *Trichoderma* as an eco-friendly biocontrol agent against *Xap* in tropical and subtropical fruit crops. Although field trials remain necessary to validate these greenhouse findings, application of *Trichoderma* to plant substrate represents a promising and sustainable management tool that aligns with societal demands for environmentally friendly agricultural practices.

## Data Availability

The original contributions presented in the study are publicly available. This data can be found here: GREDOS repository (University of Salamanca, Spain), http://hdl.handle.net/10366/172651.

## Supplementary material

Supplementary Table 1 Primers used for quantitative real-time PCR (qPCR) analysis.
