# Supplementary material for: Trichoderma controls bacterial blight caused by Xanthomonas axonopodis pv. passiflorae in Passiflora edulis
Source: Front Fungal Biol. 2026 Sep 18;7:1920680. doi: 10.3389/ffunb.2026.1920680 (PMC13630755; doi:10.3389/ffunb.2026.1920680)
Supplement: Supplementary file 1 [file Table1.docx]

**Table S1.** Primers used for quantitative real-time PCR (qPCR) analysis.

| **Gene ID** | **Forward primer (5’ → 3’)** | **Reverse primer (5’ → 3’)** | **Reference** |
| --- | --- | --- | --- |
| *LOX2* | TCTACGCCTGGGGTTACTG | TGCCTTTTGGTTTTGATGGAC | Munhoz et al., 2015 |
| *PR1* | TAGTTCTGTGCATAGGCCGC | TTACCTTGCTGCCCACAACA | Yan et al., 2021 |
| *PDF1.2* | TTGAAAACGCGTAATTAAACACAAT | TGGTTGCAACAGACCTGCTA | Yan et al., 2021 |
| *EF1* (endogenous reference gene) | GGCTGAGCGTGAACGTGGTA | CGGCACAATCAGCCTGGGAA | Wang et al., 2023 |
